# Factors Associated with Nursing Interventions for Smoking Cessation: A Narrative Review

**DOI:** 10.3390/nursrep11010007

**Published:** 2021-02-01

**Authors:** Meng Li, Keiko Koide, Miho Tanaka, Misaki Kiya, Reiko Okamoto

**Affiliations:** 1 Division of Health Sciences, Osaka University Graduate School of Medicine, Suita City 565-0871, Japan; 25b18817@sahs.med.osaka-u.ac.jp (M.L.); miho@sahs.med.osaka-u.ac.jp (M.T.); misaki_kiya@sahs.med.osaka-u.ac.jp (M.K.); 2 Faculty of Nursing, Shitennoji University, Habikino City 583-0868, Japan; keiko@shitennoji.ac.jp

**Keywords:** factors, nursing interventions, smoking cessation, narrative review

## Abstract

The purpose of this narrative review is to synthesize the factors that are associated with smoking cessation intervention among nurses. We conducted a systematic search of the literature published from database inception through to 22 April 2020, in five electronic databases including Pubmed, CINAHL Plus, Scopus, Web of science, and ProQuest. The search was limited to articles written in English and published in scientific journals. The reference lists of papers identified as being relevant in the above electronic searches were also hand searched. The initial database search yielded 2039 articles and 11 articles were obtained through a manual search. Finally, 24 articles were included in the analysis. Of the 24 included studies, 46 different factors were identified to be significantly associated with nursing interventions for smoking cessation. The identified factors were grouped into the following four conceptually similar categories: (1) socioeconomic factors, (2) smoking-related factors, (3) motivational factors, and (4) enabling factors and barriers. In the future, nursing interventions for smoking cessation will need to be improved based on the identified factors.

## 1. Introduction

The tobacco epidemic is one of the biggest public health threats the world has ever faced, resulting in more than eight million deaths a year around the world. More than seven million of those deaths are the result of direct tobacco use, while around 1.2 million are the result of non-smokers being exposed to secondhand smoke [1]. In light of this troubling fact, the international council of nurses (ICN) encourages member associations to co-ordinate their efforts with other national groups to bring government and public attention to the harmful health effects of tobacco and to encourage governments to reduce, discourage, and eliminate tobacco use, including providing access to cessation programs [2]. Nurses, representing the largest number of healthcare providers worldwide, are involved in most of these visits, and therefore have the potential for a profound effect on the reduction of tobacco use [3]. Additionally, a previous literature review reported that nurses and professional nursing organizations can make a significant difference in minimizing the disease burden caused by tobacco through nursing research, policy, practice, and education [4].

Nursing interventions for smoking cessation include various methods such as behavioral counseling for helping smokers to successfully quit smoking [5]. Regarding the effectiveness of nursing interventions for smoking cessation, the Agency for Health Care Research and Quality Clinical Practice Guideline (AHRQ) has reported that advice to stop smoking from nurses, as one of the many providers, could increase the rates of cessation [6]. The meta-analyses by Cochrane Collaboration reported that advice and support from nurses could increase people’s success to quit smoking, whether in hospitals or in community settings [7,8]. Additionally, a previous cohort study, in Japan, reported the importance of nurses’ counseling for assisting patients to achieve smoking cessation by maintaining patients’ self-efficacy of smoking cessation [9]. Overall, nursing interventions for smoking cessation play an important role to help patients quit smoking successfully.

Nurses can be more effective as the first line of treatment due to the length of time they spend with patients [10]. In addition, there are many previous studies that have reported a variety of factors associated with nursing interventions for smoking cessation; however, there is no narrative review to synthesize the factors. Therefore, the purpose of this narrative review is to synthesize these factors that are associated with nursing interventions for smoking cessation.

## 2. Materials and Methods

### 2.1. Search Strategy

We conducted a systematic search of the literature published from database inception through to 22 April 22 2020, in five electronic databases including Pubmed, CINAHL Plus, Scopus, Web of science, and ProQuest. The search was limited to articles written in English and published in scientific journals. The search was conducted using combinations of the following key words: factors, causes, influences reasons, determinants, predictors, contributors, smoking cessation, smoking cessation interventions, quit smoking, stop smoking, nursing, nurse, nursing interventions, nursing care, nursing support, nurse’s role. The detailed search strategies are presented in Supplementary Materials File S1. The reference lists of articles identified as being relevant in the above electronic searches were also hand searched.

### 2.2. Inclusion and Exclusion Criteria

Eligibility criteria were defined prior to the database search in order to only include studies that were relevant to the research question. The inclusion criteria were as follows: (a) articles written in English, (b) articles published in scientific journals, (c) quantitative studies, and (d) studies examining the factors associated with smoking cessation intervention among nurses. We excluded articles that did not meet the inclusion criteria.

### 2.3. Study Selection and Data Extraction

We examined the abstracts and full text of articles based on the predefined inclusion and exclusion criteria. Discrepancies were resolved through a consensus discussion with all researchers. The extracted data included the following items: author/year, country, study design, study subject, sample size, nursing interventions for smoking cessation, and factors significantly associated with nursing interventions for smoking cessation. Additionally, the identified factors were categorized based on the integrated-change (I-change) model [11] that demonstrated their usefulness in explaining health professionals’ behaviors related to smoking cessation interventions [12,13,14].

## 3. Results

**Figure 1:**
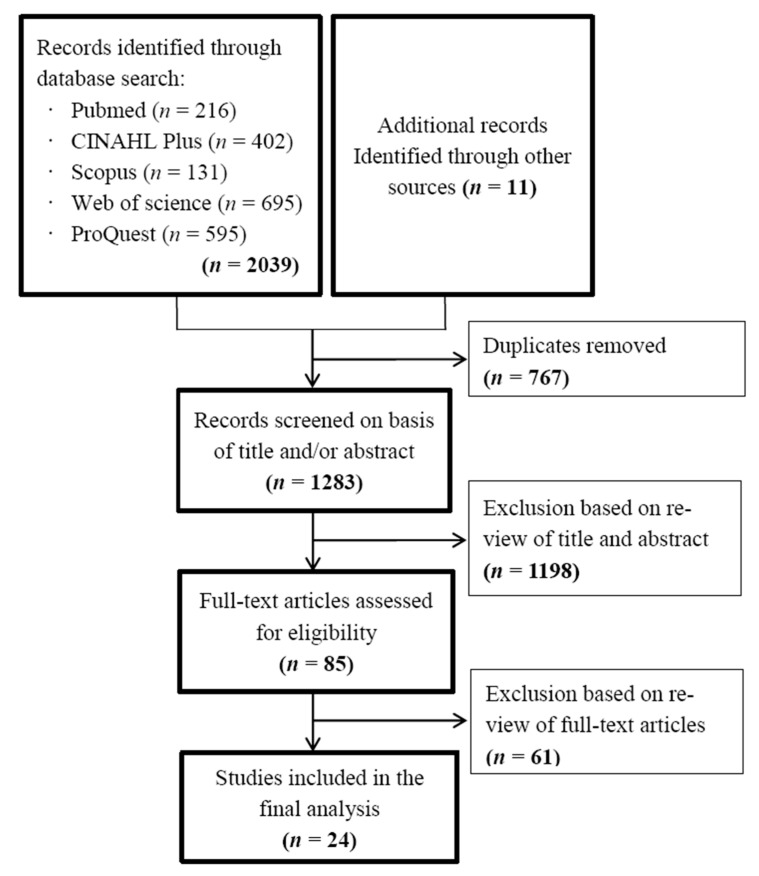
Flow chart of articles identification.

The initial database search yielded 2039 articles and 11 articles were obtained through manual search. After excluding 767 duplicate articles (automatically excluded by EndNote software, *n* = 355 and manually excluded, *n* = 412), 1283 articles remained for title and abstract screening, and then 1198 articles were excluded as they did not meet the inclusion criteria. The remaining 85 articles were subsequently assessed for full-text screening. Finally, 24 articles were included in the analysis [15,16,17,18,19,20,21,22,23,24,25,26,27,28,29,30,31,32,33,34,35,36,37,38] (Figure 1).

### 3.1. Characteristics of the Included Studies

Characteristics of the included studies are summarized in Supplementary Materials File S2. Among the 24 included articles, ten studies were conducted in the USA [15,16,25,27,28,29,30,33,37,38], three in mainland China [17,22,32], two in Hong Kong [22,24], two in the UK [21,26], and one each in Thailand [18], Australia [19], Netherlands [20], Czech Republic [31], Iceland [34], Japan [35], and Canada [36]. The design of all studies was a cross-sectional study [15,16,17,18,19,20,21,22,23,24,25,26,27,28,29,30,31,32,33,34,35,36,37,38]. The study subjects included nurses [16,17,18,20,22,23,24,25,26,28,29,30,31,32,33,34,35,36,37,38] (nurse anesthetists [38]) and midwives [15,19,21,27]. The smoking cessation intervention items were mainly developed according to the 5As (ask, advise, assess, assist, and arrange for follow-up).

### 3.2. Factors Associated with Nursing Interventions for Smoking Cessation

Of the 24 included articles, 46 different factors were identified to be significantly associated with nursing interventions for smoking cessation, and then these factors were grouped into four conceptually similar categories: (1) socioeconomic factors (gender, educational background, primary position, etc.); (2) smoking-related factors (smoking status, smoking cessation training, knowledge about smoking and quitting, etc.); (3) motivational factors (nurses’ attitude and perceptions regarding smoking and quitting, organizational support, self-efficacy, etc.); and (4) enabling factors and barriers (ability, lack of training, time and knowledge, etc.) (Table 1). Additionally, we narratively synthesized these factors that were associated with nursing interventions for smoking cessation below and more detailed narrative explanation are available in Supplementary Materials File S3.

#### 3.2.1. Sociodemographic Factors

Of the identified sociodemographic factors, we found that female nurses [22,23,24,38], more experience years [30,37], advanced position [28,29,30,37], and working in an inpatient setting [28,29,35,37] were more likely to implement smoking cessation interventions. However, age [16,23,24,27,35,37], educational background [28,29,32], and work unit [24,25,28,29,35] that were significantly associated with nursing interventions for smoking cessation were different in different studies. Additionally, academic certification [28,35], years of nursing education [35], workplaces [35], and work regions [21,23,29] were also reported to be associated with implementing smoking cessation interventions among nurses or midwifes.

#### 3.2.2. Smoking-Related Factors

Among the identified smoking-related factors, smoking status was reported to be significantly associated with implementing smoking cessation interventions among nurses or midwifes [16,23,28,29,30,31,35]. Sarna et al. (2009, 2012, and 2015) reported that nurses who were current smokers were less likely to implement smoking cessation interventions [29,30,31]. Svavarsdóttir and Hallgrímsdóttir (2007) reported that nurses, in Iceland, who were current smokers reported a significantly lower frequency of advising against smoking [35]. Obviously, nurses who were current smokers were less likely to implement smoking cessation interventions. Additionally, three previous studies reported that nurses who were ex-smokers were more likely to implement smoking cessation interventions [16,23,28].

In addition to smoking status, smoking cessation training was also reported to be significantly associated with implementing smoking cessation interventions among nurses or midwifes [17,18,24,26,37]. In China, Chan et al. (2007) reported that hospital nurses with prior training were more likely to engage in comprehensive cessation interventions, including asking patients about their use of tobacco, advising patients to quit smoking, assisting patients to attempt to quit, and arranging for follow-up contacts to prevent a relapse [17]. Chatdokmaiprai et al. (2017) reported that smoking cessation service training was positively associated with occupational health nurses’ provision of smoking cessation services in Thailand [18]. Mak et al. (2018) reported that nurses, in Hong Kong, who received the training were more likely to participate using the 5As [24]. Overall, nurses with prior smoking cessation training were more likely to implement smoking cessation interventions.

Additionally, other smoking-related factors such as knowledge regarding smoking and quitting including the harms of smoking, the benefits of quitting, and the 5As approach [23,24,38], exposure to second-hand smoke [24], and family/friend with tobacco illness [24,28] were also reported to be associated with implementing smoking cessation interventions among nurses.

#### 3.2.3. Motivational Factors

Of the identified motivational factors, four factors including attitudes or perceptions of smoking and quitting [16,21,22,23,24,25], social influence (professional norm [23,34,38], organizational support [15,18]), and self-efficacy [16,18,20,27,33,36] were reported to be associated with implementing smoking cessation interventions. Additionally, other motivational factors such as perceived motivation [16,24], as well as perceived advantages [20] and disadvantages [20,33] were also reported to be associated with implementing smoking cessation interventions among nurses or midwifes.

##### Attitudes or Perceptions on Smoking and Quitting

There were seven previous studies that reported that attitudes or perceptions about smoking and quitting were associated with smoking cessation interventions among nurses or midwives. Johnston et al. (2005) and Leung et al. (2009) reported that hospital-based registered nurses who had positive attitudes towards their own smoking cessation counseling, were likely to implement smoking cessation interventions including initiation and advice, and follow through [22,23]. Mak et al. (2018) reported that attitude about smoking and quitting was positively associated with implementing the 5As [24]. McCarty et al. (2001) reported that hospital nurses who had positive attitudes towards their role were positively associated with offering cessation advice [25]. Additionally, in the UK, Eiser et al. (1999) reported that midwives who had positive attitudes towards giving advice on smoking were more likely to implement the “warning” and “abstinence” [21]. Overall, nurses and midwives with positive attitudes or perceptions about smoking and quitting were more likely to implement smoking cessation interventions.

##### Social Influence

Among the social influence factors, the professional norm factor was reported to be significantly associated with implementing smoking cessation interventions among nurses [34,36,38]. Tremblay et al. (2009) reported that nurses’ beliefs about the role of the health professional were associated with smoking cessation counseling in Canada [36]. Yankie et al. (2006) reported that nurse anesthetists believed that smoking cessation counseling was a duty of a health care provider [38]. Svavarsdóttir & Hallgrímsdóttir (2007) reported that nurses, in Iceland, who thought smoking cessation counseling was not considered to be part of the job and was considered to be a difficult task, were less likely to implement smoking cessation counseling [34]. In addition to the professional norm factor, the organizational support factor was also reported to be significantly associated with implementing smoking cessation interventions among nurses [15,18]. Abatemarco et al. (2007) reported that the office support where the practice had a system in place to provide smoking was significantly associated with increased practice among midwives in the USA [15]. Chatdokmaiprai et al. (2017) reported that a tobacco control policy and employer support were positively related to nurses’ provision of smoking cessation services in Thailand [18]. Overall, nurses or midwives with positive professional norm and organizational support for smoking cessation intervention were more likely to implement smoking cessation interventions.

##### Self-Efficacy

There were five previous studies that reported self-efficacy was associated with smoking cessation interventions among nurses and midwives [16,18,20,27,33,36]. Chatdokmaiprai et al. (2017) reported that self-efficacy was positively related to nurses’ provision of smoking cessation services in Thailand [18]. de Ruijter et al. (2017) reported that self-efficacy significantly contributed to explaining practice nurses’ overall guideline adherence in the Netherlands [20]. Studts et al. (2010), in the USA, reported that nurse practitioners with greater self-efficacy were more likely to implement the 5As including ask, assess, assist, assist referrals and arrange follow-up [33]. Additionally, Price et al. (2006), in the USA, reported that nurse-midwives with higher outcome expectations regarding the effects of using the 5As techniques with pregnant patients who smoked were more likely to implement the 5As techniques [27]. Overall, nurses or midwives with higher self-efficacy and outcome expectations were more likely to implement smoking cessation interventions.

#### 3.2.4. Enabling Factors and Barriers

Of the identified enabling factors and barriers, lack of training, time and knowledge were huge barriers to implement smoking cessation counseling among nurses or midwifes [15,20,24,34]. Svavarsdóttir & Hallgrímsdóttir (2007) reported that lack of time, insufficient knowledge and insufficient training were associated with smoking cessation counseling among nurses in Iceland [34]. In the USA, Abatemarco et al. (2007), reported that lack of training was associated with clinical tobacco treatment practice among midwives [15]. Mak et al. (2018) reported that time availability was positively associated with the “arrange” of the 5As [24]. In addition to lack of training, time, and knowledge, previous studies have also reported that nurses or midwifes with higher self-efficacy towards engage in smoking counseling were more likely to implement smoking cessation interventions [19,22,25]. Additionally, other enabling factors and barriers such as competing priorities in the visit [15], comfort discussing cessation, comfort developing plan and comfort recommending appropriate pharmacological treatments [33], perceived patient adherence [16] were also reported to be associated with implementing smoking cessation interventions among nurses or midwifes.

## 4. Discussion

In the results, we narratively synthesized the factors that were associated with nursing interventions for smoking cessation. There are five important points that need to be discussed. First, our results indicated that nurses who were current smokers were less likely to implement smoking cessation interventions [29,30,31,35], and nurses who were ex-smokers were more likely to implement smoking cessation interventions [16,23,28]. A previous study, in Northern Ireland, reported that qualified nurses who smoked were less motivated to provide cessation support for patients, had fewer positive attitudes about the value of smoking cessation, were less likely to have received smoking cessation training, and were less likely to want further training [39]. Another previous study reported that nonsmokers and ex-smokers showed a more positive attitude toward their roles as exemplars and in counseling the public about the health hazards of smoking than smokers among oncology nurses in Texas, USA [40]. Additionally, a systematic review with meta-analysis reported that nurses’ personal smoking status was not significantly associated with nurses always asking patients about their smoking, assessing motivation and assisting patients to quit smoking, but nurses who smoked were 13% less likely to advise their patients to quit and 25% were less likely to arrange smoking cessation follow-up [41]. Overall, support and assistance for nurses to quit smoking are needed to strengthen nursing interventions for smoking cessation.

Secondly, our results indicate that nurses with prior smoking cessation training were more likely to implement smoking cessation interventions [17,18,24]. A previous study, in the Czech Republic, reported that nurses’ brief intervention skills including asking patients about smoking, recommendations to stop smoking, assessing willingness to quit, assisting with cessation, and recommending a smoke-free home were significantly improved after the completion of an e-learning program [42]. Additionally, two previous studies reported that nurses receiving web-based smoking cessation education significantly increased self-reports of frequency of providing interventions to patients who smoked, including recommending smoke-free home environments to support attempts to quit [43,44]. The meta-analyses by Cochrane Collaboration reported that healthcare professionals who had received training were more likely to perform tasks of smoking cessation [45]. Therefore, providing smoking cessation training can improve smoking cessation intervention skills, and then implement nursing interventions for smoking cessation.

Thirdly, our results indicate that nurses with positive attitudes [21,22,23,24,25] and social influence [15,18,34,36,38] for smoking cessation intervention were more likely to implement smoking cessation interventions. A previous focus group study reported that there is a need to build upon nurses’ positive attitudes about engaging in smoking cessation interventions with patients to ensure that cessation interventions are standard nursing practice [46]. Additionally, previous studies reported that social influence towards smoking cessation intervention had a significant positive influence on determining the intention to implement smoking cessation intervention [12,14]. Overall, positive attitudes and social influence regarding smoking cessation interventions can promote nursing interventions for smoking cessation.

Fourthly, our results indicate that nurses with higher self-efficacy and outcome expectations were more likely to implement smoking cessation interventions [16,18,20,27,33,36]. A previous integrative review of the literature reported that attitude, innovation, perceived social influence, and self-efficacy were factors for occupational health nurses’ intention to implement smoking cessation interventions; therefore, improving occupational health nurses’ self-efficacy could guide changes in clinical practice for motivating smokers to quit [47]. Regarding the strategies to improve nurses’ attitudes and self-efficacy, a previous study, in India, reported that adequate experience in a center for addiction medicine improved nurses’ positive attitude and self-efficacy, and therefore helped to provider substantial care to patients with addiction problem [48]. Therefore, improving nurses’ self-efficacy is needed to strengthen nursing interventions for smoking cessation.

Finally, our results also indicate that nurses with higher self-efficacy to engage in smoking counseling were more likely to implement smoking cessation interventions [19,22,25]. A previous study, in Japan, reported that research utilization competency was positively associated with self-efficacy and prenatal smoking cessation interventions among public health nurses [49]. Additionally, another study including 1054 primary healthcare nurses, in Sweden, reported that the ability and use of research were significant determinants of attitudes towards research and use of research findings [50]. Overall, nurses with higher research utilization ability were more likely to implement smoking cessation interventions.

There were two limitations of this review. One was that these factors might not be applicable to all nurses because the characteristics of nurses such as the types of nurse, work location, and work position were different. Another limitation was that the search produced only articles published in English and in selected databases. Thus, this might have missed other studies that were published in other languages and were in other databases.

## 5. Conclusions

In this review, we narratively synthesized the factors that were associated with nursing interventions for smoking cessation. There were 46 different factors identified as significantly associated with nursing interventions for smoking cessation. The identified factors were grouped into the following four conceptually similar categories: (1) socioeconomic factors, (2) smoking-related factors, (3) motivational factors, and (4) enabling factors and barriers. In the future, nursing interventions for smoking cessation will need to be improved based on the identified factors.

## Figures and Tables

**Table 1:** Summary of factors significantly associated with nursing interventions for smoking cessation.

Categories	Factors Significantly Associated with Nursing Interventions	Author Year (Reference Number)
Sociodemographic factors	Gender	Johnston et al. (2005) [22], Leung et al. (2009) [23], Mak et al. (2018) [24], Yankie et al. (2006) [38].
	Age	Wetta-Hall et al. (2005) [37], Price et al. (2006) [27], Mak et al. (2018) [24], Leung et al. (2009) [23], Taniguchi et al. (2011) [35], Borrelli et al. (2001) [16]
	Work experience	Wetta-Hall et al. (2005) [37], Sarna et al. (2012) [30]
	Educational background	Sarna et al. (2000) [28], Sarna et al. (2009) [29], Sarna et al. (2016) [32]
	Primary position	Sarna et al. (2000) [28], Sarna et al. (2009) [29], Sarna et al. (2012) [30], Wetta-Hall et al. (2005) [37]
	Primary work setting	Sarna et al. (2000) [28], Sarna et al. (2009) [29], Taniguchi et al. (2011) [35], Wetta-Hall et al. (2005) [37]
	Work unit	Sarna et al. (2000) [28], McCarty et al. (2001) [25], Sarna et al. (2009) [29], Taniguchi et al. (2011) [35], Mak et al. (2018) [24]
	Academic certification	Taniguchi et al. (2011) [35], Sarna et al. (2000) [28]
	Level of nursing education	Taniguchi et al. (2011) [35]
	Workplace type	Taniguchi et al. (2011) [35]
	Region	Eiser et al. (1999) [21], Leung et al. (2009) [23]
	State of residence	Sarna et al. (2009) [29]
Smoking-related factors	Smoking status	Sarna et al. (2009) [29], Sarna et al. (2012) [30], Sarna et al. (2015) [31], Svavarsdóttir & Hallgrímsdóttir (2007) [35], Leung et al. (2009) [23], Sarna et al. (2000) [28], Borrelli et al. (2001) [16]
	Smoking cessation training	Chan et al. (2007) [17], Chatdokmaiprai et al. (2017) [18], Mak et al. (2018) [24], McEwen et al. (2001) [26], Wetta-Hall et al. (2005) [37]
	Knowledge on smoking and quitting	Leung et al. (2009) [23], Mak et al. (2018) [24], Yankie et al. (2006) [38]
	Exposed to second-hand smoke	Mak et al. (2018) [24]
	Family/friend suffering from smoking-related diseases	Mak et al. (2018) [24], Sarna et al. (2000) [28]
	Familiar with Tobacco Free Nurses	Sarna et al. )2009) [29]
	Clinical practice guideline awareness	Studts et al. (2010) [33], Yankie et al. (2006) [38]
	Implementing the 5As including “ask” “advise”, “assess”, “assist” and “arrange”	Sarna et al. (2012) [30]
	Perceived severity of tobacco health consequences	Studts et al. (2010) [33]
	Pharmacotherapy for smoking cessation	Studts et al. (2010) [33]
Motivational factors	Nurses’ attitude and perceptions on smoking and quitting	Borrelli et al. (2001) [16], de Ruijter et al. (2017) [20], Eiser et al. (1999) [21], Johnston et al. (2005) [22], Leung et al. 2009 [23], Mak et al. (2018) [24], McCarty et al. (2001) [25]
	Organizational support	Abatemarco et al. (2007) [15], Chatdokmaiprai et al. (2017) [18], Cooke et al. (1996) [19].
	Self-efficacy	Borrelli et al. (2001) [16], Chatdokmaiprai et al. (2017) [18], de Ruijter et al. (2017) [20], Studts et al. (2010) [33], Tremblay et al. 2009 [36].
	Outcome expectations	Borrelli et al. (2001) [16], Price et al. (2006) [27]
	Professional norm	Leung et al. (2009) [23], Tremblay et al. (2009) [36], Yankie et al. (2006) [38], Svavarsdóttir & Hallgrímsdóttir 2007 [34].
	Perceived motivation for smokers	Borrelli et al. (2001) [16], Mak et al. (2018) [24]
	Perceived efficacy expectations	Price et al. (2006) [27]
	Response efficacy–cessation	Studts et al. (2010) [33]
	Response efficacy–brief	Studts et al. (2010) [33]
	Perceived effectiveness	Borrelli et al. (2001) [16]
	Counseling is worthwhile	Yankie et al. (2006) [38]
	Interaction between the man and not a duty	Yankie et al. (2006) [38]
	Nurses who wanted to receive training	Mak et al. (2018) [24]
Enabling factors and barriers	Ability	Cooke et al. (1996) [19], Johnston et al. (2005) [22], McCarty et al. (2001) [25]
	Lack of training, time and knowledge	Abatemarco et al. (2007) [15], Svavarsdóttir & Hallgrímsdóttir (2007) [34], de Ruijter et al. (2017) [20], Mak et al. (2018) [24]
	Competing priorities in the visit	Abatemarco et al. (2007) [15]
	Perceived patient adherence	Borrelli et al. (2001) [16]
	Cohesion, work pressure and clarity	Cooke et al. (1996) [19]
	Comfort discussing cessation	Studts et al. (2010) [33]
	Comfort developing plan	Studts et al. (2010) [33]
	Comfort recommending appropriate pharmacological treatments	Studts et al. (2010) [33]
	Perceived barriers	Studts et al. (2010) [33]
	Only advise if patient asks for information	McCarty et al. (2001) [25]
	Possessing skills	Wetta-Hall et al. (2005) [37]

## Data Availability

No Data Available.

## Supplementary Materials

The following are available online at https://www.mdpi.com/2039-4403/11/1/7/s1, Supplementary File S1: Detailed search strategies, Supplementary File S2: Characteristics of the included studies, Supplementary File S3: Detailed narrative explanation on factors associated with nursing interventions for smoking cessation.
