# Supplementary material for: Factors Associated with Nursing Interventions for Smoking Cessation: A Narrative Review
Source: Nurs Rep. 2021 Feb 1;11(1):64–74. doi: 10.3390/nursrep11010007 (PMC8608102; doi:10.3390/nursrep11010007)
Supplement: Supplementary file 1 [file nursrep-11-00007-s001.zip › S1-final.docx]

Supplementary File S1: Detailed search strategies

**Keywords:** factors or causes or influences or reasons or determinants or predictors or contributors AND　smoking cessation or smoking cessation interventions or quit smoking or stop smoking AND　nursing or nurse or nursing interventions or nursing care or nursing support or nurse's role

**Language:** English
**Search date:** April 22, 2020

A systematic search was conducted with the following databases.

**CiNAHL**

(((factors[Title/Abstract] OR causes[Title/Abstract] OR influences[Title/Abstract] OR reasons[Title/Abstract] OR determinants[Title/Abstract] OR predictors[Title/Abstract] OR contributors[Title/Abstract])) AND (smoking cessation[Title/Abstract] OR smoking cessation interventions[Title/Abstract] OR quit smoking[Title/Abstract] OR stop smoking[Title/Abstract])) AND (nursing[Title/Abstract] OR nurse[Title/Abstract] OR nursing interventions[Title/Abstract] OR nursing care[Title/Abstract] OR nursing support[Title/Abstract] OR nurse's role[Title/Abstract])

**Timespan:** 1983-2020

**Results:** 402 records

**Pubmed**

Search: (((factors[Title/Abstract] OR causes[Title/Abstract] OR influences[Title/Abstract] OR reasons[Title/Abstract] OR determinants[Title/Abstract] OR predictors[Title/Abstract] OR contributors[Title/Abstract])) AND (smoking cessation[Title/Abstract] OR smoking cessation interventions[Title/Abstract] OR quit smoking[Title/Abstract] OR stop smoking[Title/Abstract])) AND (nursing[Title/Abstract] OR nurse[Title/Abstract] OR nursing interventions[Title/Abstract] OR nursing care[Title/Abstract] OR nursing support[Title/Abstract] OR nurse's role[Title/Abstract])

**Timespan:** -April 22, 2020

**Results:** 216 records

**Web of science**

(factors or causes or influences or reasons or determinants or predictors or contributors) AND TOPIC: (smoking cessation or smoking cessation interventions or quit smoking or stop smoking) AND TOPIC: (nursing or nurse or nursing interventions or nursing care or nursing support or nurse's role)

**Timespan:** All years.

**Results:** 695 records

**Scopus**

( TITLE-ABS-KEY ( factors OR causes OR influences OR reasons OR determinants OR predictors OR contributors ) AND TITLE-ABS-KEY ( smoking AND cessation OR smoking AND cessation AND interventions OR quit AND smoking OR stop AND smoking ) AND TITLE-ABS-KEY ( nursing OR nurse OR nursing AND interventions OR nursing AND care OR nursing AND support OR nurse's AND role ) )

**Timespan:** All years

**Results:** 131 records

**ProQuest**

ab(factors OR causes OR influences OR reasons OR determinants OR predictors OR contributors) AND ab(smoking cessation OR smoking cessation interventions OR quit smoking OR stop smoking) AND ab(nursing OR nurse OR nursing interventions OR nursing care OR nursing support OR nurse's role)

**Timespan:** All years

**Results:** 595 records
