# Supplementary material for: Factors Associated with Nursing Interventions for Smoking Cessation: A Narrative Review
Source: Nurs Rep. 2021 Feb 1;11(1):64–74. doi: 10.3390/nursrep11010007 (PMC8608102; doi:10.3390/nursrep11010007)
Supplement: Supplementary file 1 [file nursrep-11-00007-s001.zip › S2-final.docx]

Supplementary File S2: Characteristics of the included studies

| **Author/year** | **Country** | **Study design** | **Study subject** | **Sample**  **size** | **Nursing interventions for smoking cessation** | **Factors significantly associated with nursing interventions for smoking cessation** |
| --- | --- | --- | --- | --- | --- | --- |
| [Abatemarco](https://www.ncbi.nlm.nih.gov/pubmed/?term=Abatemarco%20DJ%5bAuthor%5d&cauthor=true&cauthor_uid=17826707) et al.  2007 [15] | USA |  | Midwives | 193 | Eleven specific tobacco treatment items which include the 5As:   1. Ask patients if they smoke; 2. Ask patients if they live with a smoker; 3. Ask smokers how much they smoke; 4. Explain dangers of tobacco use; 5. Advise smokers to stop smoking; 6. Ask smokers if they are interested in quitting; 7. Encourage them to set a quit date; 8. Discuss medication options (i.e., nicotine replacement or bupropion SR); 9. Follow-up with a letter, call or visit about their smoking; 10. Refer for smoking cessation treatment; 11. Reevaluate tobacco use at each visit. | Lack of training, competing priorities in the visit (e.g., acute illness), whether the practice had a system in place to provide smoking cessation information and resources. |
| [Borrelli](https://www.ncbi.nlm.nih.gov/pubmed/?term=Borrelli B%5BAuthor%5D&cauthor=true&cauthor_uid=11701297) et al.  2001 [16] | USA | Cross-sectional study | Home healthcare nurses | 98 | 1. The amount of time spent discussing tobacco use. 2. Nurse’s style of discussing tobacco use: 3. I discussed smoking only if the clients brought up the subject or appeared motivated to quit; 4. I discussed smoking only if the clients had a smoking-related health problem; 5. I made it a point to discuss smoking with all of my clients who smoke, regardless of health status or interest in quitting; 6. Did not discuss smoking.   3. 4As-Ask, Advise, Assist, and Arrange  (1) Ask patients about their smoking status;  (2) Advise patients to quit smoking;  (3) Assist patients who wanted to stop smoking by providing referrals and advice to quit;  (4) Arrange a follow-up visit or phone call to discuss quitting.  4. Nurse recommendations:  (1) Encourage their patients to stop smoking completely and permanently;  (2) Encourage their patients to use nicotine replacement;  (3) Encourage their patients to reduce smoking to five or fewer cigarettes per day, if the patient stated that they could not quit;  (4) Encourage their patients not to smoke in the presence of infants and children. | **Time Spent Discussing Tobacco Use:** outcome expectations;  **Nurse’s style of discussing tobacco use:** self-efficacy;  **Ask, Advise and Assist:** nurses’ perception of the importance of smoking counseling;  **Advise:** perceived effectiveness, smoking status;  **Assist:** perceived patient adherence, smoking status; **Arrange:** self-efficacy (among former or nonsmoking nurses).  **Recommend “cutting down” to patients:** Age, perceived patient motivation;  **Recommend the use of nicotine replacement:** perceived greater health risks of smoking, perceived effectiveness. |
| [Chan](https://www.ncbi.nlm.nih.gov/pubmed/?term=Chan%20SS%5bAuthor%5d&cauthor=true&cauthor_uid=17393965) et al.  2007 [17] | China | Cross-sectional study | Hospital nurses | 1690 | Ask:   1. Assess smoking history and status for all patients; 2. Assess level of nicotine dependence; 3. Record encounter in nursing Kardex (e.g., smoking status, intervention/counseling given, quit date proposed, etc);   Advise:   1. Explain dangers of smoking; 2. Explain dangers of passive smoking; 3. Advice to stop smoking; 4. Motivate patients’ intention to quit smoking; 5. Explain to patients how smoking may have contributed to their illness;   Assess:   1. Help patient identify triggers to smoke; 2. Assess patient’s readiness to quit smoking;   Assist:   1. Help patient develop a cessation plan, including a quit date; 2. Refer patients to other resources as appropriate; 3. Offer self-help materials for quitting where possible; 4. Suggest specific strategies for patients to quit or reduce cigarettes; 5. Recommend alternatives to smoking (e.g., exercise); 6. Recommend nicotine replacement therapy; 7. With patient’s consent, discuss his/her smoking with family members; 8. Refer patient to other health care professionals, e.g., patient’s physician for smoking cessation counseling; 9. Organize seminars and health talks on tobacco and health; 10. Refer patient to other health care professionals, e.g., patient’s physician, for smoking-cessation counseling; 11. Organize seminars and health talks on tobacco and health;   Arrange   1. Arrange follow-up for smokers; 2. Teach coping skills to prevent relapse; 3. Encourage relapsed smokers to try quitting again. | **5As:** prior training in smoking cessation interventions. |
| [Chatdokmaiprai](https://www.ncbi.nlm.nih.gov/pubmed/?term=Chatdokmaiprai%20K%5BAuthor%5D&cauthor=true&cauthor_uid=28075716) et al. 2017 [18] | Thailand | Cross-sectional study | Occupational  health nurses | 254 | Smoking cessation interventions (i.e., 5As), with 10-item scale. | Tobacco control policy, employer support, smoking cessation services training, and self-efficacy. |
| [Cooke](https://pubmed.ncbi.nlm.nih.gov/?term=Cooke+M&cauthor_id=8972929) et al.  1996 [19] | Australia | Cross-sectional study | Midwives | 425 | Assessment:   1. Assessment of smoking; 2. Stickers to indicate smoking status;   Education:   1. Education about risk; 2. Pamphlet on smoking effects; 3. Video on smoking;   Advice:   1. Advice to cut down; 2. Advice to quit;   Counselling:   1. Methods to quit; 2. Encourage support person to assist; 3. Follow-up discussion; 4. Self-help quit booklet; 5. Negotiate quit date;   Referral:   1. Quit group; 2. Drug counsellor; 3. Health promotion counsellor. | **Assessment:** ability;  **Assessment, Smoking advice, Education and Counselling:** policy, funding and size (structural predictors), cohesion, work pressure and clarity (work climate predictor). |
| [de Ruijter](https://www.ncbi.nlm.nih.gov/pubmed/?term=de%20Ruijter%20D%5BAuthor%5D&cauthor=true&cauthor_uid=28486612) et al. 2017 [20] | Netherlands | Cross-sectional study | Practice nurses | 157 | Overall guideline adherence:   1. Offer a quit advice; 2. Assess smoking profile and smoking history; 3. Assess motivation to quit; 4. Increasing motivation; 5. Assess barriers to quitting; 6. Discuss barriers; 7. Inform about cessation aids; 8. make a quit plan and setting a quit date; 9. Arrange follow-up after the quit date. | **Overall guideline adherence:** perceived advantages, practice nurses’ self-efficacy, time spent on counselling;  **(3) - (9)**: practice nurses’ self-efficacy; **(5) and (6)**: time spent on counselling; **(6)**: perceived advantages;  **(7)**: perceived disadvantages. |
| [Eiser](https://psycnet.apa.org/search/results?term=Eiser,%20J.%20Richard&latSearchType=a) et al. 1999 [21] | UK | Cross-sectional study | Midwives | 138 | Facilitation:   1. For those who are not sure if they want to give up, discuss worries about giving up; 2. Discuss why it may be difficult for the mother to stop; 3. Mention the cost of smoking; 4. Advise partners who smoke to try and cut down; 5. Advise those women who smoke during pregnancy to stop completely; 6. Explain the benefits of giving up; 7. Advise partners who smoke to stop completely; 8. For those who have given up recently, discuss how to avoid going back; 9. Ask partners of pregnant women if they smoke; 10. Suggest that the woman gives up with another smoker if possible;   Warning:   1. Discuss the effect of passive smoking on the new born child; 2. Offer ongoing support/encouragement; 3. Discuss general dangers of smoking to the unborn baby; 4. Check whether the woman understands the various bits of advice; 5. Give out a leaflet on smoking; 6. Discuss general dangers of smoking to the mother; 7. Record smoking status in the women's records; 8. After delivery, advise the woman to stop smoking in the presence of the baby;   Abstinence:   1. Advise women who smoke to try and cut down; 2. Arrange with the women a date on which to stop smoking for good when you next see; 3. Say that you will ask about smoking the woman; 4. Warn about the danger of having an occasional cigarette;   (23) Discuss tobacco withdrawal symptoms. | **Facilitation and warning:** region;  **Warning and abstinence:** role attitudes (among never smokers). |
| [Johnston](https://pubmed.ncbi.nlm.nih.gov/?term=Johnston+JM&cauthor_id=15530592) et al. 2005 [22] | Hong Kong | Cross-sectional survey | Hospital -based registered  nurses | 1843 | Ask about smoking:   1. Assess smoking history and status for all smoking clients;   Advise smokers to quit smoking:   1. Explain the dangers of smoking; 2. Explain the dangers of passive or second-hand smoking; 3. Advise to stop smoking; 4. Motivate clients’ intention to quit; 5. Explain to clients how smoking may have contributed to their illness;   Assess the readiness to quit   1. Help smoking client identify his/her triggers to smoke; 2. Assess smoking patients' readiness to quit smoking;   Assist patients to stop smoking:   1. Refer smoking clients to resources as appropriate (e.g. smoking cessation clinic); 2. Suggest specific actions that clients could quit or cut down their smoking activity; 3. Recommend alternatives to smoking; 4. With smoking clients' consent, discuss his/her smoking with family members; 5. Refer to other health care professionals; 6. Organize seminars/health talks on tobacco and health;   Arrange follow up:   1. Teach coping skills to prevent relapse where appropriate; 2. Encourage relapsed smokers to try quitting again. | **Initiation and advice:** attitude to own smoking cessation counseling;  **Follow-through:** gender, attitude to own smoking cessation counseling, self-perceived competence in smoking cessation counseling. |
| Leung et al.  2009 [23] | China | Cross-sectional study | Hospital -based registered  nurses | Guangzhou: 1541  Hong Kong: 1843. | Ask about smoking:   1. Assess smoking history and status for all smoking clients; 2. Assess level of nicotine dependence;   Advise smokers to quit smoking:   1. Explain the dangers of smoking; 2. Explain the dangers of passive or second-hand smoking;   Assess the readiness to quit   1. Help smoking client identify his/her triggers to smoke; 2. Assess smoking patients' readiness to quit smoking;   Assist patients to stop smoking:   1. Help motivational smoking patient develop a cessation plan, including a quit date; 2. Refer smoking clients to resources as appropriate (e.g. smoking cessation clinic); 3. Offer self-help materials for quitting to patients who smoke; 4. Reinforce smoking clients' intention to quit smoking; 5. Suggest specific actions that clients could do to make quitting or cutting; down easier, e.g., distraction, drinking water, avoiding social gathering, etc.; 6. Organize seminars/health talks on tobacco and health; 7. With smoking clients' consent, discuss his/her smoking with family members;   Arrange follow up:   1. Arrange follow-up specifically for smokers; 2. Teach coping skills to prevent relapse where appropriate; 3. Encourage relapsed smokers to try quitting again. | **Initiation and advice:** general knowledge, specific knowledge, attitudes towards tobacco promotion, nurse's own responsibility in smoking cessation;  **Follow-through:** gender, age (26-30 years), specific knowledge and attitudes towards tobacco promotion (Hongkong).  **Initiation and advice:** smoking status, specific knowledge;  **Follow-through:** age (31-35 years), smoking status, specific knowledge, attitudes towards tobacco promotion (Guangzhou). |
| [Mak](https://www.ncbi.nlm.nih.gov/pubmed/?term=Mak%20YW%5bAuthor%5d&cauthor=true&cauthor_uid=29789484) et al. 2018 [24] | Hong Kong | Cross-sectional study | Registered nurses | 4413 | 1. Ask about the smoking status of service users; 2. Advise smokers to quit smoking; 3. Assess the readiness of smokers to quit; 4. Assist smokers in quitting smoking; 5. Refer smokers to smoking-cessation services. | **Advise and Assist: g**ender;  **Ask, Advise, Assist and Arrange:** age; **Ask, Advise, Assess, Assist and Arrange**: want to receive training, training received in smoking cessation interventions, primary area of work, attitude on smoking and quitting; **Assess**: family members suffering from smoking-related diseases;  **Ask, Advise and Assist**: exposed to second-hand smoke;  **Advise, Assess and Assist**: **s**mokers’ motivation to quit;  **Ask, Advise, Assess and Arrange**: health benefits of quitting smoking; **Ask and Arrange:** time availability; **Advise, Assess and Arrange**: knowledge on smoking and quitting. |
| [McCarty](https://pubmed.ncbi.nlm.nih.gov/?term=McCarty+MC&cauthor_id=11716666) et al.  2001 [25] | USA | Cross-sectional study | Hospital nurses | 397 | Advise patients who smoke cigarettes to stop smoking. | Attitudes toward offering smoking cessation advice, ability to provide smoking cessation advice, work unit, Only advise if patient asks for information. |
| [McEwen](https://pubmed.ncbi.nlm.nih.gov/?term=McEwen+A&cauthor_id=11226357) et al.  2001 [26] | UK | Cross-sectional study | Practice nurses | 459 | Advise patients to stop smoking:   1. Advise smokers to stop during most or all consultations; 2. Advise smokers to stop during consultations every now and then; 3. Advise smokers to cut down if they are unwilling or unable to stop; 4. Advise smokers to stop at least every now and then;   Assistance:   1. given out leaflets on how to stop smoking; 2. lent out videos on how to stop smoking; 3. Provide counselling yourself to smokers wanting to stop; 4. Run a stop smoking group;   Referral:   1. Refer patients to a hypnotherapist; 2. Refer patients to an acupuncturist; 3. Refer patients to another alternative therapist; 4. Refer patients to any form of alternative therapy; 5. Recommend that patients go to a private smokers clinic; 6. Referred patients to a NHS/research smokers clinic; 7. Advise patients to phone Quitline.   Nurses recommending:   1. Recommend that patients go and buy nicotine gum; 2. Recommend that patients go and buy the nicotine patch; 3. Recommend that patients go and buy the nicotine inhalator; 4. Recommend some form of NRT. | Receive training. |
| [Price](https://www.sciencedirect.com/science/article/abs/pii/S1526952305006616#!) et al. 2006 [27] | USA | Cross-sectional study | Nurse-midwives | 194 | 5As:   1. Documents cigarette smoking use status at each visit; 2. Give clear, strong advice to quit with a personalized message about the impact of smoking on the unborn child; 3. Assess whether the patient is willing to make a quit attempt within the next 30 days; 4. Assist by encouraging the use of problem-solving skills for smoking cessation; 5. Assist by providing and/or arranging for social support to help her stop smoking; 6. Provide self-help smoking cessation materials to pregnant smokers; 7. Use counseling to help patients willing to make a quit attempt; 8. Prescribe nicotine replacement therapy to help patients willing to make a quit attempt; 9. Schedule follow-up contact, in person or by telephone, within the first week after the quit date for patients with whom you establish a quit smoking date; 10. Refer patients willing to make a quit attempt to outside agencies who conduct smoking cessation. | Age, perceived efficacy expectations, outcome expectations. |
| [Sarna](https://www.ncbi.nlm.nih.gov/pubmed/?term=Sarna%20LP%5BAuthor%5D&cauthor=true&cauthor_uid=10951353) et al. 2000 [28] | USA | Cross-sectional study | Oncology nurses | 1508 | 1. Assess tobacco use; 2. Document tobacco use; 3. Assess readiness to quit; 4. Provide counseling about tobacco use; 5. Provide cessation advice; 6. Teach coping skills to prevent relapse; 7. Recommend nicotine replacement; 8. Refer to a smoking cessation specialist; 9. Refer to another practitioner; 10. Recommend a support group for cessation; 11. Provide information about second-hand Smoke; 12. Provide information to family members; 13. Assist other nurses with quitting smoking. | **Assess tobacco status and Document on chart**: primary position, work setting, primary practice setting;  **Assess readiness to quit**: work setting;  **Give advice:** smoking history and current status, family/friend with tobacco illness, educational background, primary position, work setting;  **Provides counseling**: family/friend with tobacco illness, certification, primary position, work setting; **Recommend nicotine replacement therapy**: family/friend with tobacco illness, educational background, work setting;  **Teach skills to prevent relapse**: smoking history and current status, family/friend with tobacco illness, primary position. |
| Sarna et al. 2009 [29] | USA | Cross-sectional study | Nurses | 4489 | 5As:   1. Ask about tobacco use; 2. Advise to quit; 3. Assess readiness to quit; 4. Assist with quit efforts, including recommendations of pharmacotherapy; 5. Arrange for follow-up; 6. Recommend medications; 7. Refer to Quitline; 8. Refer to community resource. | **Advise, Assess, Assist, Arrange, Recommend medications, Refer to Quitline and Refer to community resource:** familiar with Tobacco Free Nurses;  **Arrange:** smoking status;  **Ask and Advise**: level of nursing education;  **Advise, Arrange, Recommend medications, Refer to Quitline and Refer to community resource:** primary position;  **5As, Recommend medications, Refer to Quitline and Refer to community resource:** unit where the nurse usually worked;  **Advise, Assess, Assist, Arrange, Refer to Quitline and Refer to community resource:** state of residence**.** |
| [Sarna](https://www.ncbi.nlm.nih.gov/pubmed/?term=Sarna%20LP%5BAuthor%5D&cauthor=true&cauthor_uid=10951353) et al. 2012 [30] | USA | Cross-sectional study | Nurses | 2437 | 5As:   1. Ask; 2. Advise; 3. Assess; 4. Assist; 5. Arrange for follow-up; 6. Refer to Quitline. | **Advise, Assess, Assist, Arrange and Refer to Quitline:** tobacco use state; **Advise, Arrange and Refer to Quitline**: smoking status;  **Ask, Advise and Assist:** years of practice;  **Ask and Refer to Quitline:** professional level;  **Refer to Quitline:** advise, assess, assist, arrange, tobacco use state. |
| [Sarna](https://www.ncbi.nlm.nih.gov/pubmed/?term=Sarna%20LP%5BAuthor%5D&cauthor=true&cauthor_uid=10951353) et al. 2015 [31] | Czech Republic | Cross-sectional study | Nurses | 157 | 5As:   1. Ask about smoking/tobacco use; 2. Advise patients to quit smoking; 3. Assess readiness to quit smoking; 4. Assist with smoking cessation 5. Arrange smoking cessation follow-up 6. Recommend the telephone quitline; 7. Refer to community cessation resources 8. Recommend tobacco cessation medications; 9. Review barriers to quitting; 10. Recommend creating a smoke-free home environment. | **Assess, Arrange, Recommend the telephone quitline and Recommend smoke-free home**: smoking status. |
| [Sarna](https://www.ncbi.nlm.nih.gov/pubmed/?term=Sarna%20LP%5BAuthor%5D&cauthor=true&cauthor_uid=10951353) et al. 2016 [32] | China | Cross-sectional study | Registered nurses | 2440 | 5As:   1. Ask about patient’s smoking/tobacco use; 2. Advise patients to quit smoking; 3. Assess readiness to quit; 4. Assist with smoking cessation; 5. Arrange smoking cessation follow-up; 6. Refer to tobacco cessation resources (classes, clinics, counselling, etc.) in the community; 7. Refer to tobacco cessation resources (classes, clinics, counselling, etc.) in the community; 8. Recommend tobacco cessation medications; 9. Review barriers to quitting; 10. Recommend reducing exposure to second-hand smoke; 11. Referral to the quitline (only Beijing). | **Referral to the quitline**: education (only Beijing). |
| Studts et al. 2010 [33] | USA | Cross-sectional study | Nurse practitioners | 193 | 5As:   1. Ask every patient to identify tobacco use status, 2. How tobacco use status is determined; 3. Frequency of asking; 4. Advise patients to quit; 5. Assess tobacco user’s willingness to quit using tobacco 6. Assist tobacco users to quit using tobacco; 7. Referrals to cessation resources; 8. Types of follow-up. | **Ask, Advise, Assess, Assist, Assist referrals and Arrange follow-up:** clinical practice guideline awareness;  **Assess, Assist and Arrange follow-up:** pharmacotherapy;  **Ask, Advise, Assess, Assist and Arrange follow-up:** comfort discussing cessation;  **Advise, Assess, Assist, Assist referrals and Arrange follow-up:** comfort developing plan;  **Assess, Assist and Arrange follow-up:** comfort recommending appropriate pharmacological treatments;  **Ask:** perceived severity;  **Ask, Assess, Assist, Assist referrals and Arrange follow-up:** self-efficacy;  **Ask and Arrange follow-up:** response efficacy–cessation;  **Advise and Assist:** response efficacy–brief;  **Advise and Assess:** perceived barriers. |
| [Svavarsdóttir](https://www.ncbi.nlm.nih.gov/pubmed/?term=Svavarsd%C3%B3ttir%20MH%5BAuthor%5D&cauthor=true&cauthor_uid=17419789) & [Hallgrímsdóttir](https://www.ncbi.nlm.nih.gov/pubmed/?term=Hallgr%C3%ADmsd%C3%B3ttir%20G%5BAuthor%5D&cauthor=true&cauthor_uid=17419789). 2007 [34] | Iceland | Cross-sectional study | Nurses | 868 | 5As:   1. Discuss the health gain of smoking cessation; 2. Discuss the risks of smoking; 3. Discuss ways to prevent relapse; 4. Inform about abstinence symptoms; 5. Recommend nicotine replacement therapy or bupropion; 6. Brochure; 7. Refer to a quit smoking phone line; 8. Refer to a smoking cessation course; 9. Decide a quit date; 10. Provide a follow-up visit or a phone call. | **5As:** lack of time, insufficient knowledge, insufficient training, not considered a part of job and considered difficult task.  **Advise**: nurses’ smoking status. |
| [Taniguchi](https://www.ncbi.nlm.nih.gov/pubmed/?term=Taniguchi%20C%5BAuthor%5D&cauthor=true&cauthor_uid=21821967) et al. 2011[35] | Japan | Cross-sectional study | Nurses | 2215 | 1. Tobacco use assessment and documentation; 2. Cessation advice; Assessment of readiness to quit; 3. Individualized explanation of harmful effects of tobacco use; 4. Arrangements for enrollment in a smoking cessation program. | **Tobacco use assessment and documentation**: age, length of nursing education and current work setting type;  **Cessation advice and Individualized explanation of harmful effects of tobacco use:** current work division; **Tobacco use assessment and documentation, Assessment of readiness to quit and Individualized explanation of harmful effects of tobacco use:** academic certification;  **Tobacco use assessment and documentation; Cessation advice; Assessment of readiness to quit; Individualized explanation of harmful effects of tobacco use:** workplace type. |
| [Tremblay](https://www.ncbi.nlm.nih.gov/pubmed/?term=Tremblay%20M%5BAuthor%5D&cauthor=true&cauthor_uid=19770488) et al. 2009 [36] | Canada | Cross-sectional study | Nurses | 251 | 5As:   1. Ask about smoking status; 2. Advise smokers to quit; 3. Assess readiness to quit; 4. Assist smokers ready to quit; 5. Refer smokers ready to quit; 6. Arrange appointment 1-2 weeks after quit date for smokers ready to quit; 7. Assist smokers not ready to quit; 8. Arrange follow-up for smokers not ready to quit. | **“Ready to quit” counseling**: beliefs about the role of the health professional, self-efficacy;  **“Not ready to quit” counseling**: beliefs about the role of the health professional. |
| Wetta-Hall et al. 2005 [37] | USA | Cross-sectional study | Office-based nurses | 290 | 1. Tobacco use assessment; 2. Patient interest in tobacco cessation assessed; 3. Tobacco cessation advice given. | **Tobacco use assessment and Tobacco cessation advice given**: type of medical practice;  **Tobacco use assessment and Tobacco cessation advice given**: age, type of nurse;  **Tobacco use assessment, Tobacco cessation advice given and Tobacco cessation advice given**: having skills is a facilitator, cessation continued education in the past years;  **Tobacco use assessment**: years experience. |
| [Yankie](https://www.ncbi.nlm.nih.gov/pubmed/?term=Yankie%20VM%5BAuthor%5D&cauthor=true&cauthor_uid=16546015) et al. 2006 [38] | USA | Cross-sectional study | Nurse anesthetists | 276 | 1. General advice to quit; 2. Provide health risk information; 3. Benefits of quitting; 4. Suggest pharmacologic agents; 5. Discuss obstacles to quitting; 6. Referral to a support group; 7. Cold turkey method; 8. Tapering method; 9. Setting a quit date; 10. Provide brochure or literature. | Gender; knowledge on the ‘5 A’s’ approach; duty as health care provider; counseling is worthwhile; aware of USPHS guideline; interaction between the man and not a duty. |
