# Supplementary material for: Factors Associated with Nursing Interventions for Smoking Cessation: A Narrative Review
Source: Nurs Rep. 2021 Feb 1;11(1):64–74. doi: 10.3390/nursrep11010007 (PMC8608102; doi:10.3390/nursrep11010007)
Supplement: Supplementary file 1 [file nursrep-11-00007-s001.zip › S3-final.docx]

Supplementary File S3: Detailed narrative explanation on factors associated with nursing interventions for smoking cessation.

Factors associated with nursing interventions for smoking cessation: A narrative review

3.2.1. Sociodemographic factors

3.2.1.1. Gender

There were four previous studies that reported gender was associated with smoking cessation interventions among nurses and midwives. [Johnston](https://pubmed.ncbi.nlm.nih.gov/?term=Johnston+JM&cauthor_id=15530592) et al. (2005) and Leung et al. (2009) reported that female nurses were more likely to offer “follow-through” activities (frequent or occasional engagement in assessing or assisting with or arranging smoking cessation activities) than male nurses in hospitals of Hong Kong [22,23]. [Mak](https://www.ncbi.nlm.nih.gov/pubmed/?term=Mak%20YW%5bAuthor%5d&cauthor=true&cauthor_uid=29789484) et al. (2018) reported that female nurses were more likely to advise and assist to quit smoking in Hong Kong [24]. [Yankie](https://www.ncbi.nlm.nih.gov/pubmed/?term=Yankie%20VM%5BAuthor%5D&cauthor=true&cauthor_uid=16546015) et al. (2006) reported that the certified registered nurse anesthetists who were men were less likely than women to provide counseling for their patients in the USA [38]. Overall, female nurses were more likely to implement the smoking cessation interventions than male.

3.2.1.2. Age

There were five previous studies that reported age was associated with smoking cessation interventions among nurses and midwives. Wetta-Hall et al. (2005) reported that older office-based nurses aged 41–50 were more likely to assess tobacco use and younger middle-aged nurses aged 31–40 were more likely to assess a patient’s interest in stopping tobacco use in Kansas, USA [37]. Price et al. (2006) reported that there was a significant but modest positive correlation between the use of the 5As model and older age of nurse-midwives in Ohio, USA [27]. Mak et al. (2018) reported that more mature nurses were more likely to participate in providing advice and assistance and in making arrangements in Hong Kong [24]. However, Leung et al. (2009) reported that nurses aged 26–30 were less likely than nurses aged 21–25 to offer “follow-through” activities in the Hong Kong sample, while nurses aged 31–35 were less likely than nurses aged 21–25 to implement “follow-through” activities in the Guangzhou, China [23]. [Taniguchi](https://www.ncbi.nlm.nih.gov/pubmed/?term=Taniguchi%20C%5BAuthor%5D&cauthor=true&cauthor_uid=21821967) et al. (2011) reported that hospital nurses aged 20–29 were more likely to implement tobacco use assessment and documentation than nurses aged over 30 [35]. [Borrelli](https://www.ncbi.nlm.nih.gov/pubmed/?term=Borrelli%20B%5BAuthor%5D&cauthor=true&cauthor_uid=11701297) et al. (2001) reported that older nurses were less likely to recommend “cutting down” to patients who were not motivated to quit [16]. Overall, age that was significantly associated with smoking cessation interventions was different in different studies.

3.2.1.3. Years of work experience

There were two previous studies that reported years of work experience were associated with smoking cessation interventions among nurses and midwives. Wetta-Hall et al. (2005) reported that office-based nurses were less likely to assess patient tobacco use if they had practiced as a nurse for less than or equal to five years [37]. Sarna et al. (2012) reported that nurses with less than 15 years of practice were more likely to consistently Ask, Advise, Assess and Assist smokers to quit, but not Arrange or Refer to a Quitline in the USA [30]. Overall, nurses with more experience years were more likely to implement the smoking cessation interventions.

3.2.1.4. Educational background

There were three previous studies that reported educational background was associated with smoking cessation interventions among nurses and midwives. Sarna et al. (2000) reported that oncology nurses who had college education were more likely to recommend nicotine replacement therapy and less likely to give advice in the USA [28]. Sarna et al. (2009) reported that nurses who were masters/doctors were more likely to ask about tobacco use and advise to quit than nurses who were bachelors, and nurses who were bachelors were more likely to ask about tobacco use than licensed practice nurses in the USA [29]. Sarna et al. (2016) reported that diploma educated nurses more likely to refer to the quitline than nurses with an associate or baccalaureate degree in Beijing, China [32]. Overall, educational background that was significantly associated with smoking cessation interventions was different in different studies.

3.2.1.5. Primary position

There were four previous studies that reported primary position was associated with smoking cessation interventions among nurses and midwives. Sarna et al. (2000) reported that nurse practitioners were more likely to document on chart, provide counseling and teach skills to prevent relapse, and staff nurses were less likely to give advice and teach skills to prevent relapse in the USA [28]. Sarna et al. (2009) reported that advanced practice nurses were more likely to advise, arrange, recommend medications, refer to Quitline and refer to community resource in the USA [29]. Sarna et al. (2012) reported that advanced practice nurses were more likely to ask and refer to Quitline [30]. Wetta-Hall et al. (2005) reported that office-based nurse practitioners were more likely to preform tobacco use assessment and assessment of patient Interest in smoking cessation in Kansas, USA [37]. Overall, nurses with advanced position were less likely to implement smoking cessation interventions.

3.2.1.6. Primary work setting

There were four previous studies that reported primary work setting was associated with smoking cessation interventions among nurses and midwives. Sarna et al. (2000) reported that nurses in inpatient setting were more likely to assess tobacco status, document on chart and recommend nicotine replacement therapy, whereas nurses in inpatient setting were less likely to assess readiness to quit, give advice and provide counseling [28]. Sarna et al. (2009) reported that nurses working in outpatient facilities were less likely to offer assistance with cessation [29]. [Taniguchi](https://www.ncbi.nlm.nih.gov/pubmed/?term=Taniguchi%20C%5BAuthor%5D&cauthor=true&cauthor_uid=21821967) et al. (2011) reported that hospital nurses in inpatient care were more likely to perform tobacco use assessments and documentation in Japan [35]. Wetta-Hall et al. (2005) reported that nurses in an internal medicine office or family practice office were more likely to assess tobacco use and give tobacco cessation advice, whereas nurses in rural office setting were less likely to give tobacco cessation advice [37]. Overall, nurses in inpatient setting were more likely to implement smoking cessation interventions.

3.2.1.7. Work unit

There were five previous studies that reported work unit was associated with smoking cessation interventions among nurses and midwives. Sarna et al. (2000) reported that nurses in oncology setting were more likely to assess tobacco status and document on chart [28]. [McCarty](https://pubmed.ncbi.nlm.nih.gov/?term=McCarty+MC&cauthor_id=11716666) et al. (2001) reported that nurses in cardiology units were more likely to offer advice [25]. Sarna et al. (2009) reported that compared with nurses working in medical-surgical units, nurses in intensive care units were more likely to intervene. Nurses working in obstetrical/gynecological units (OB/GYN) and emergency rooms were more likely to ask patients about their tobacco use, but less likely to offer cessation interventions [29]. [Taniguchi](https://www.ncbi.nlm.nih.gov/pubmed/?term=Taniguchi%20C%5BAuthor%5D&cauthor=true&cauthor_uid=21821967) et al. (2011) reported that hospital nurses in a surgical setting were more likely to provide cessation advice and providing individualized explanation of the harmful effects of tobacco use [35]. Mak et al. (2018) reported that nurses who worked primarily in the Medicine Department and the Ambulatory/Outpatient Department were more likely to ask, advise, assess, assist and arrange for follow-up in Hong Kong [24]. Overall, work unit that was significantly associated with smoking cessation interventions was different in different studies.

3.2.1.8. Others

[Taniguchi](https://www.ncbi.nlm.nih.gov/pubmed/?term=Taniguchi%20C%5BAuthor%5D&cauthor=true&cauthor_uid=21821967) et al. (2011) reported that nurses with an academic certification (Certifified by Japan Nursing Association or other academic society) were more likely to assess readiness to quit and perform individualized explanation of harmful effects of tobacco use than those without such certification, whereas nurses with an academic certification were less likely to perform tobacco use assessment and documentation than those without such certification in Japan [35].

[Sarna](https://www.ncbi.nlm.nih.gov/pubmed/?term=Sarna%20LP%5BAuthor%5D&cauthor=true&cauthor_uid=10951353) et al. (2000) reported that certified nurses were more likely to provides counseling [28].

[Taniguchi](https://www.ncbi.nlm.nih.gov/pubmed/?term=Taniguchi%20C%5BAuthor%5D&cauthor=true&cauthor_uid=21821967) et al. (2011) reported that nurses with 4 years or more of nursing education were more likely to perform tobacco use assessment and documentation than nurses with less than 4 years of nursing education [35].

[Taniguchi](https://www.ncbi.nlm.nih.gov/pubmed/?term=Taniguchi%20C%5BAuthor%5D&cauthor=true&cauthor_uid=21821967) et al. (2011) reported that nurses working in designated cancer hospital were more likely to perform tobacco use assessment and documentation, cessation advice, assessment of readiness to quit and individualized explanation of harmful effects of tobacco use [35].

Sarna et al. (2009) reported that nurses working in hospitals in states with high tobacco prevalence also were significantly more likely to deliver cessation interventions on a more frequent basis [29].

[Eiser](https://psycnet.apa.org/search/results?term=Eiser,%20J.%20Richard&latSearchType=a) et al. (1999) reported that regions in the UK were significantly associated with advising to quit smoking for pregnant smokers among midwifes [21].

Leung et al. (2009) reported that regions (Hongkong and Guangzhou) in China were significantly associated with levels of practice in all the five strategies in smoking cessation [23].

3.2.2. Smoking-related factors

3.2.2.1. Smoking status

There were six previous studies that reported smoking status was associated with smoking cessation interventions among nurses and midwives. Sarna et al. (2009) reported that nurses who were current smokers were less likely to arrange for follow-up in the USA [29]. Sarna et al. (2012) reported that nurses who were current smokers were less likely to consistently Advise, Arrange, and Refer smokers to a Quitline compared to never/former smokers in the USA [30]. Sarna et al. (2015) reported that nurses who were current smokers had the lowest frequency of assessing readiness to quit, arranging for follow-up, referring to a quitline, or recommending a smoke-free environment in the Czech Republic [31]. [Svavarsdóttir](https://www.ncbi.nlm.nih.gov/pubmed/?term=Svavarsd%C3%B3ttir%20MH%5BAuthor%5D&cauthor=true&cauthor_uid=17419789) & [Hallgrímsdóttir](https://www.ncbi.nlm.nih.gov/pubmed/?term=Hallgr%C3%ADmsd%C3%B3ttir%20G%5BAuthor%5D&cauthor=true&cauthor_uid=17419789). (2007) reported that nurses who were current smokers reported a significantly lower frequency of advising against smoking [35]. In addition, Leung et al. (2009) reported that hospital nurses who were ex-smokers were more likely to provide both "initiation and advice" and "follow-through" activities than never smokers in Guangzhou, China [23]. Sarna et al. (2000) reported that oncology nurses who were ex-smokers were more likely to give advice and teach skills to prevent relapse [28]. [Borrelli](https://www.ncbi.nlm.nih.gov/pubmed/?term=Borrelli%20B%5BAuthor%5D&cauthor=true&cauthor_uid=11701297) et al. (2001) reported that current smokers were 89% less likely to advise their patients to quit than never- or ex-smokers and 81% less likely to insist that patients do not smoke in front of their children [16]. Overall, nurses who were current smokers were less likely to implement smoking cessation interventions, whereas nurses who were ex-smokers were more likely to implement smoking cessation interventions.

3.2.2.2. Smoking cessation training

There were five previous studies that reported smoking cessation training was associated with smoking cessation interventions among nurses and midwives. [Chan](https://www.ncbi.nlm.nih.gov/pubmed/?term=Chan%20SS%5bAuthor%5d&cauthor=true&cauthor_uid=17393965) et al. (2007) reported that hospital nurses with prior training were more likely to engage in comprehensive cessation interventions, including asking patients about the use of tobacco, advising patients to quit smoking, assisting patients to make a quit attempt, and arranging for follow-up contacts to prevent relapse in China [17]. [Chatdokmaiprai](https://www.ncbi.nlm.nih.gov/pubmed/?term=Chatdokmaiprai%20K%5BAuthor%5D&cauthor=true&cauthor_uid=28075716) et al. (2017) reported that smoking cessation services training were positively associated with occupational health nurses’ provision of smoking cessation services in Thailand [18]. Mak et al. (2018) reported that nurses who received the training were more likely to participate on the 5As in Hongkong [24]. [McEwen](https://pubmed.ncbi.nlm.nih.gov/?term=McEwen+A&cauthor_id=11226357) et al. (2001) reported that practice nurses who reported having received training in the giving of smoking cessation advice were more likely than those who had not to say that they provided assistance for smokers wanting to stop (counselling, leaflets, etc). In particular they were more likely to say that they provided counselling for smokers wanting to stop, handed out leaflets, ran a stop smoking group, and received referrals from general practitioners in the practice. The practice nurses with training were also more likely to report that their practice owned a carbon monoxide monitor. Practice nurses who had been trained in smoking cessation were more likely to advise patients to telephone the Quitline, and less likely to refer smokers to an alternative therapist. They were also more likely to believe that if a smoker changes from high to low nicotine cigarettes the risk of lung cancer stays the same. Practice nurses with training were more likely to report recommending NRT, to believe that NRT is effective enough to justify its cost, and to believe that NRT should be on general sale [26]. Wetta-Hall et al. (2005) reported that office-based nurses who had no tobacco-related continuing education in the previous year were less likely to assess tobacco use, patient’s interest in stopping smoking and give tobacco cessation advice [37]. Overall, nurses with prior smoking cessation training were more likely to implement smoking cessation interventions.

3.2.2.3. Knowledge on smoking and quitting

There were three previous studies that reported knowledge on smoking and quitting was associated with smoking cessation interventions among nurses and midwives. Leung et al. (2009) reported that the specific knowledge in smoking-related diseases was positively associated with both "initiation and advice" and "follow-through" interventions for hospital nurses in Hong Kong and Guangzhou, China, and the general knowledge about the impact of smoking on health were positively associated with the "initiation and advice" smoking cessation intervention in Hong Kong [23]. Mak et al. (2018) reported that knowledge of the harms of smoking and the benefits of quitting was positively associated with advise, assess, and arrange in Hongkong [24]. [Yankie](https://www.ncbi.nlm.nih.gov/pubmed/?term=Yankie VM%5BAuthor%5D&cauthor=true&cauthor_uid=16546015) et al. (2006) reported that nurse anesthetists with knowledge on the ‘5 A’s’ approach was significantly more likely to provide smoking cessation counseling to their patients than others in the USA [38]. Overall, nurses with knowledge on smoking and quitting were more likely to implement smoking cessation interventions.

3.2.2.4. Others

[Sarna](https://www.ncbi.nlm.nih.gov/pubmed/?term=Sarna%20LP%5BAuthor%5D&cauthor=true&cauthor_uid=10951353) et al. (2009) reported that familiarity with Tobacco Free Nurses (TFN) was associated with smoking cessation intervention in the USA [29].

Studts et al. (2010) reported that clinical practice guideline awareness was associated with clinical practices regarding treatment of tobacco use and dependence among nurse practitioners in the US [33].

[Yankie](https://www.ncbi.nlm.nih.gov/pubmed/?term=Yankie%20VM%5BAuthor%5D&cauthor=true&cauthor_uid=16546015) et al. (2006) reported that aware of the US Public Health Service’s (USPHS) Clinical Practice was associated with smoking cessation counseling among nurse anesthetists in the US [38].

Mak et al. (2018) reported that nurses who had family members who suffered from smoking-related diseases were more likely to participate in making assessments in Hongkong [24].

Mak et al. (2018) reported that nurses who had been exposed to second-hand smoke were more likely to participate in asking, advising, and assisting in Hongkong [24].

[Sarna](https://www.ncbi.nlm.nih.gov/pubmed/?term=Sarna%20LP%5BAuthor%5D&cauthor=true&cauthor_uid=10951353) et al. (2000) reported that certified nurses who had family/friend with tobacco illness were more likely to give advice, provides counseling, recommend nicotine replacement therapy and Teach skills to prevent relapse in the USA [28].

[Sarna](https://www.ncbi.nlm.nih.gov/pubmed/?term=Sarna%20LP%5BAuthor%5D&cauthor=true&cauthor_uid=10951353) et al. (2012) reported that nurses with implementing the “advise”, “assess”, “assist” and “arrange” were associated with the “Refer to Quitline” [30].

Studts et al. (2010) reported that pharmacotherapy for smoking cessation was associated with “Assess”, “Assist” and “Arrange follow-up” among nurse practitioners in the US [33].

Studts et al. (2010) reported that perceived severity of tobacco health consequences was associated with “Ask” among nurse practitioners in the US [33].

3.2.3. Motivational factors

3.2.3.1. Nurses’ attitudes and perceptions on smoking and quitting

There were seven previous studies that reported nurses’ attitudes and perceptions on smoking and quitting were associated with smoking cessation interventions among nurses and midwives. [Borrelli](https://www.ncbi.nlm.nih.gov/pubmed/?term=Borrelli%20B%5BAuthor%5D&cauthor=true&cauthor_uid=11701297) et al. (2001) reported that nurses who had positive perceptions (general risk perception and patient risk perception) on the importance of smoking counseling was more likely to ask, advise and assist, and nurses who perceived greater health risks of smoking were significantly more likely to recommend the use of nicotine replacement to their patients in the USA [16]. [de Ruijter](https://www.ncbi.nlm.nih.gov/pubmed/?term=de%20Ruijter%20D%5BAuthor%5D&cauthor=true&cauthor_uid=28486612) et al. (2017) reported that nurses who had perceived advantages were more likely to implement overall guideline adherence and more perceived disadvantages were more likely to inform about cessation aids in the Netherlands [20]. [Eiser](https://psycnet.apa.org/search/results?term=Eiser,%20J.%20Richard&latSearchType=a) et al. (1999) reported that midwives who had positive attitudes towards giving advice on smoking were more likely to implement the “warning” and “abstinence” in the UK [21]. [Johnston](https://pubmed.ncbi.nlm.nih.gov/?term=Johnston+JM&cauthor_id=15530592) et al. (2005) reported that hospital -based registered nurses who had positive attitude to own smoking cessation counseling were likely to implement initiation and advice, and follow-through in Hongkong [22]. Leung et al. (2009) reported that hospital -based registered nurses who had positive attitude towards tobacco promotion were likely to implement initiation and advice, and follow-through in Hongkong and nurses who had positive attitude to own smoking cessation counseling were likely to implement follow-through in Guangzhou [23]. [Mak](https://www.ncbi.nlm.nih.gov/pubmed/?term=Mak%20YW%5bAuthor%5d&cauthor=true&cauthor_uid=29789484) et al. (2018) reported that the attitude on smoking and quitting was positively associated with implementing the 5As [24]. [McCarty](https://pubmed.ncbi.nlm.nih.gov/?term=McCarty+MC&cauthor_id=11716666) et al. (2001) reported that hospital nurses who had positive attitude toward their role were positively associated with offering cessation advice [25]. Overall, nurses with positive attitudes and perceptions on smoking and quitting were more likely to implement smoking cessation interventions.

3.2.3.2.Organizational. [support](C:/Program%20Files/Youdao/Dict/8.9.3.0/resultui/html/index.html#/javascript:;) for smoking cessation intervention

There were three previous studies that reported [organizational](C:/Program%20Files/Youdao/Dict/8.9.3.0/resultui/html/index.html#/javascript:;) [support](C:/Program%20Files/Youdao/Dict/8.9.3.0/resultui/html/index.html#/javascript:;) for smoking cessation intervention was associated with smoking cessation interventions among nurses and midwives. [Abatemarco](https://www.ncbi.nlm.nih.gov/pubmed/?term=Abatemarco%20DJ%5bAuthor%5d&cauthor=true&cauthor_uid=17826707) et al. (2007) reported that the office support whether the practice had a system in place to provide smoking was significantly associated with increased practice among midwives in the US [15]. [Chatdokmaiprai](https://www.ncbi.nlm.nih.gov/pubmed/?term=Chatdokmaiprai%20K%5BAuthor%5D&cauthor=true&cauthor_uid=28075716) et al. (2017) reported that tobacco control policy and employer support were positively related to nurses’ provision of smoking cessation services in Thailand [18]. [Cooke](https://pubmed.ncbi.nlm.nih.gov/?term=Cooke+M&cauthor_id=8972929) et al. (1996) reported that midwives who worked in larger hospitals (> 300 beds) and in hospitals which had a smoking intervention policy were more likely to implement smoking intervention [19]. Overall, nurses with [organizational](C:/Program%20Files/Youdao/Dict/8.9.3.0/resultui/html/index.html#/javascript:;) [support](C:/Program%20Files/Youdao/Dict/8.9.3.0/resultui/html/index.html#/javascript:;) for smoking cessation intervention were more likely to implement smoking cessation interventions.

3.2.3.2. Self-efficacy for smoking cessation intervention

There were five previous studies that reported self-efficacy for smoking cessation intervention was associated with smoking cessation interventions among nurses and midwives. [Borrelli](https://www.ncbi.nlm.nih.gov/pubmed/?term=Borrelli%20B%5BAuthor%5D&cauthor=true&cauthor_uid=11701297) et al. (2001) reported that home healthcare nurses’ self-efficacy (confidence in their own ability to counsel patients about smoking) was significantly positively associated with the style of discussing tobacco use that nurses would discuss tobacco use with their patients regardless of their health status, and among former or nonsmoking nurses, self-efficacy was a borderline significant predictor of arranging follow-up in the US [16]. [Chatdokmaiprai](https://www.ncbi.nlm.nih.gov/pubmed/?term=Chatdokmaiprai%20K%5BAuthor%5D&cauthor=true&cauthor_uid=28075716) et al. (2017) reported that self-efficacy was positively related to nurses’ provision of smoking cessation services in Thailand [18]. [de Ruijter](https://www.ncbi.nlm.nih.gov/pubmed/?term=de%20Ruijter%20D%5BAuthor%5D&cauthor=true&cauthor_uid=28486612) et al. (2017) reported that self-efficacy significantly contributed to explaining practice nurses’ overall guideline adherence in the Netherlands [20]. Studts et al. (2010) reported that nurse practitioners with greater self-efficacy were more likely to implement ask, assess, assist, assist referrals and arrange follow-up in the US [33]. [Tremblay](https://www.ncbi.nlm.nih.gov/pubmed/?term=Tremblay%20M%5BAuthor%5D&cauthor=true&cauthor_uid=19770488) et al. (2009) reported that self-efficacy was positively associated with the “Ready to quit” counseling among nurses in Canada [36]. Overall, nurses with higher self-efficacy were more likely to implement smoking cessation interventions.

3.2.3.3. Outcome expectations

There were two previous studies that reported outcome expectations was associated with smoking cessation interventions among nurses and midwives. [Borrelli](https://www.ncbi.nlm.nih.gov/pubmed/?term=Borrelli%20B%5BAuthor%5D&cauthor=true&cauthor_uid=11701297) et al. (2001) reported that home healthcare nurses with high outcome expectations (the nurse’s perception of the effectiveness of smoking-cessation counseling in helping their patients to successfully quit) were more likely to spend more time in discussing tobacco use in the US [16]. [Price](https://www.sciencedirect.com/science/article/abs/pii/S1526952305006616#!) et al. (2006) reported that nurse-midwives with higher outcome expectations regarding the effects of using the 5A’s techniques with pregnant patients who smoked were more likely to implement the 5A’s techniques in the US [27]. Overall, nurses with higher outcome expectations were more likely to implement smoking cessation interventions.

3.2.3.4. Professional norm

There were four previous studies that reported professional norm was associated with smoking cessation interventions among nurses and midwives. Leung et al. (2009) reported that hospital -based registered nurses’ attitude towards their own responsibility in smoking cessation were positively associated with the "initiation and advice" smoking cessation intervention in Hong Kong [23]. [Tremblay](https://www.ncbi.nlm.nih.gov/pubmed/?term=Tremblay%20M%5BAuthor%5D&cauthor=true&cauthor_uid=19770488) et al. (2009) reported that nurses’ beliefs about the role of the health professional were associated with smoking cessation counseling in Canada [36]. [Yankie](https://www.ncbi.nlm.nih.gov/pubmed/?term=Yankie%20VM%5BAuthor%5D&cauthor=true&cauthor_uid=16546015) et al. (2006) reported that nurse anesthetists who believe smoking cessation counselling as a duty as health care provider [38]. However, [Svavarsdóttir](https://www.ncbi.nlm.nih.gov/pubmed/?term=Svavarsd%C3%B3ttir%20MH%5BAuthor%5D&cauthor=true&cauthor_uid=17419789) & [Hallgrímsdóttir](https://www.ncbi.nlm.nih.gov/pubmed/?term=Hallgr%C3%ADmsd%C3%B3ttir%20G%5BAuthor%5D&cauthor=true&cauthor_uid=17419789). (2007) reported that nurses who think smoking cessation counselling was not considered a part of job and it was considered difficult task, were less likely to implement the smoking cessation counselling in Iceland [34]. Overall, nurses with positive professional norm were more likely to implement smoking cessation interventions.

3.2.3.5. Perceived motivation for smokers

There were two previous studies that reported professional norm was associated with smoking cessation interventions among nurses and midwives. [Borrelli](https://www.ncbi.nlm.nih.gov/pubmed/?term=Borrelli%20B%5BAuthor%5D&cauthor=true&cauthor_uid=11701297) et al. (2001) reported that perceived patient motivation was related to recommend “cutting down” to patients” in the US [16]. [Mak](https://www.ncbi.nlm.nih.gov/pubmed/?term=Mak%20YW%5bAuthor%5d&cauthor=true&cauthor_uid=29789484) et al. (2018) reported that smokers’ motivation to quit was associated with “Advise”, “Assess” and “Assist” in Hongkong [24]. Overall, perceived motivation can encourage smokers to quit smoking.

3.2.3.6. Others

[Price](https://www.sciencedirect.com/science/article/abs/pii/S1526952305006616#!) et al. (2006) reported that nurse-midwives with higher perceived efficacy expectations in their ability to communicate issues about the 5As were more likely to implement the 5A’s techniques in the US [27].

Studts et al. (2010) reported that nurse practitioners with higher response efficacy regarding tobacco cessation in general were more likely to implement the “Ask” and “Arrange follow-up” [33].

Studts et al. (2010) reported that nurse practitioners with higher response efficacy regarding brief interventions were more likely to implement the “Advise” and “Assist” in the US [33].

[Borrelli](https://www.ncbi.nlm.nih.gov/pubmed/?term=Borrelli%20B%5BAuthor%5D&cauthor=true&cauthor_uid=11701297) et al. (2001) reported that home healthcare nurses who believed that their counseling (perceived effectiveness) would be effective in helping patients quit were more likely to advise their patients to quit and to recommend the use of nicotine replacement to their patients in the US [16].

[Yankie](https://www.ncbi.nlm.nih.gov/pubmed/?term=Yankie%20VM%5BAuthor%5D&cauthor=true&cauthor_uid=16546015) et al. (2006) reported that nurse anesthetists who think counseling is worthwhile was more likely to provide smoking cessation counseling [38].

[Yankie](https://www.ncbi.nlm.nih.gov/pubmed/?term=Yankie%20VM%5BAuthor%5D&cauthor=true&cauthor_uid=16546015) et al. (2006) reported that nurse anesthetists who think man was not a duty for smoking cessation were negatively associated with providing smoking cessation counseling [38].

Mak et al. (2018) reported that nurses who wanted to receive training in smoking-cessation interventions were more likely to participate on the 5As [24].

3.2.4. Enabling factors and barriers

3.2.4.1. Ability

There were three previous studies that reported ability was associated with smoking cessation interventions among nurses and midwives. [Cooke](https://pubmed.ncbi.nlm.nih.gov/?term=Cooke+M&cauthor_id=8972929) et al (1996) reported that midwives with higher ability to engage in smoking counselling were likely to implement the “Assessment” in Australia [19]. [Johnston](https://pubmed.ncbi.nlm.nih.gov/?term=Johnston+JM&cauthor_id=15530592) et al. (2005) reported that self-perceived competence in smoking cessation counseling was positively associated with offering follow-through interventions in Hongkong [22]. [McCarty](https://pubmed.ncbi.nlm.nih.gov/?term=McCarty+MC&cauthor_id=11716666) et al. (2001) reported that hospital nurses with higher ability to provide smoking cessation advice were positively associated with offering cessation advice in the US [25]. Overall, nurses with higher ability were more likely to implement smoking cessation interventions.

3.2.4.2. Lack of training, time and knowledge

There were four previous studies that reported ability was associated with smoking cessation interventions among nurses and midwives. [Abatemarco](https://www.ncbi.nlm.nih.gov/pubmed/?term=Abatemarco DJ[Author]&cauthor=true&cauthor_uid=17826707) et al. (2007) reported that lack of training was associated with clinical tobacco treatment practice among midwives in the US [15]. [Svavarsdóttir](https://www.ncbi.nlm.nih.gov/pubmed/?term=Svavarsd%C3%B3ttir%20MH%5BAuthor%5D&cauthor=true&cauthor_uid=17419789) & [Hallgrímsdóttir](https://www.ncbi.nlm.nih.gov/pubmed/?term=Hallgr%C3%ADmsd%C3%B3ttir%20G%5BAuthor%5D&cauthor=true&cauthor_uid=17419789). (2007) reported that lack of time, insufficient knowledge and insufficient training were associated with smoking cessation counselling among nurses in Iceland [34]. [de Ruijter](https://www.ncbi.nlm.nih.gov/pubmed/?term=de%20Ruijter%20D%5BAuthor%5D&cauthor=true&cauthor_uid=28486612) et al. (2017) reported that the amount of time spent on counselling was found to significantly correlate with smoking cessation treatment guideline adherence [20]. [Mak](https://www.ncbi.nlm.nih.gov/pubmed/?term=Mak%20YW%5bAuthor%5d&cauthor=true&cauthor_uid=29789484) et al. (2018) reported that time availability was positively associated with the “Arrange” of the 5As [24]. Overall, lack of training, time and knowledge were huge barriers to implement smoking cessation counselling among nurses.

3.2.4.3. Others

[Abatemarco](https://www.ncbi.nlm.nih.gov/pubmed/?term=Abatemarco%20DJ%5bAuthor%5d&cauthor=true&cauthor_uid=17826707) et al. (2007) reported that competing priorities in the visit (e.g., acute illness) was associated with clinical tobacco treatment practice among midwives in the US [15].

[Borrelli](https://www.ncbi.nlm.nih.gov/pubmed/?term=Borrelli%20B%5BAuthor%5D&cauthor=true&cauthor_uid=11701297) et al. (2001) reported that perceived patient adherence was associated with the “Assist” [16].

[Cooke](https://pubmed.ncbi.nlm.nih.gov/?term=Cooke+M&cauthor_id=8972929) et al. (1996) reported that cohesion, work pressure and clarity (work climate predictor) were associated with “Assessment”, “Smoking advice”, and “Education and Counselling” [19].

Studts et al. (2010) reported that comfort discussing cessation, comfort developing plan and comfort recommending appropriate pharmacological treatments were associated with clinical practices regarding treatment of tobacco use and dependence among nurse practitioners in the US [33].

Studts et al. (2010) reported that perceived barriers to tobacco cessation treatment practices was associated with “Ask” among nurse practitioners in the US [33].

[McCarty](https://pubmed.ncbi.nlm.nih.gov/?term=McCarty+MC&cauthor_id=11716666) et al. (2001) reported that hospital nurses who was the type of “I only advise patients to quit smoking when they ask me for information about smoking cessation” was negatively associated with offering cessation advice in the US [25].

Wetta-Hall et al. (2005) reported that office-based nurses with possessing skills to support patient tobacco cessation efforts was perceived as a facilitator to implement “tobacco use assessment”, “tobacco cessation advice given” and “tobacco cessation advice given” [37].
